# *MsTHI1* overexpression improves drought tolerance in transgenic alfalfa (*Medicago sativa* L.)

**DOI:** 10.3389/fpls.2022.992024

**Published:** 2022-09-08

**Authors:** Hang Yin, Zhaoyu Wang, Han Li, Yu Zhang, Mei Yang, Guowen Cui, Pan Zhang

**Affiliations:** Department of Grassland Science, College of Animal Science and Technology, Northeast Agricultural University, Harbin, China

**Keywords:** alfalfa, *MsTHI1*, photosynthesis, antioxidant defense, osmotic homeostasis, drought tolerance

## Abstract

In recent years, drought stress caused by global warming has become a major constraint on agriculture. The thiamine thiazole synthase (THI1) is responsible for controlling thiamine production in plants displaying a response to various abiotic stresses. Nonetheless, most of the THI1 activities in plants remain largely unknown. In this study, we extracted *MsTHI1* from alfalfa and demonstrated its beneficial impact on improving the resistance of plants to stress conditions. The highest levels of *MsTHI1* expression were identified in alfalfa leaves, triggered by exposure to cold, drought, salt, or alkaline conditions. The upregulation of *MsTHI1* in drought-stressed transgenic plants resulted in enhanced accumulation of vitamin B1 (VB1), chlorophyll *a* (Chl *a*), chlorophyll *b* (Chl *b*), soluble protein, higher soil and plant analyzer development (SPAD) value, and the activity of peroxidase (POD), maintained Fv/Fm, and decreased lipid peroxidation. Moreover, overexpression of *MsTHI1* upregulated the transcription of *THI4, TPK1, RbcX2, Cu/Zn-SOD, CPK13*, and *CPK32* and downregulated the transcription of *TH1* and *CPK17* in transgenic alfalfa under drought stress. These results suggested that *MsTHI1* enhances drought tolerance by strengthening photosynthesis, regulating the antioxidant defense system, maintaining osmotic homeostasis, and mediating plant signal transduction.

## Introduction

Plants must deal with abiotic and biotic stresses in the wild, where they are constantly subjected to unfavorable environmental stresses (Zhu, 2016; Rao et al., 2020). The increased frequency and intensity of extreme weather events due to climate changes make drought stress a significant constraint on agriculture (Rivero et al., 2022). Climate change has made drought an even more substantial problem, dramatically reducing crop yields and quality and limiting plant development (Li et al., 2020). Plants respond to drought conditions by altering photosynthetic, physiological, metabolic, and molecular activities, which in turn causes membrane rearrangement, osmotic pressure aggravation, the buildup of reactive oxygen species (ROS), in addition to functional abnormalities in cellular components (Roo et al., 2020; Yan et al., 2020). As a result, plants have developed sophisticated defense mechanisms to mitigate the detriment of drought conditions, from molecular and physiological to cellular and ecological levels (Xu and Zhou, 2008; Hussain et al., 2018). Rapid genetic enhancements in drought-tolerant crops may be possible if proteins or genes involved in such alterations are identified.

As an enzyme cofactor, thiamine [vitamin B1 (VB1)] is an integral part of the diets of all living species, since it is required for the appropriate metabolic activity of all living things (Strobbe et al., 2022). In plants, the biosynthesis of thiamine is promoted during adaptation responses to persistent abiotic stresses, such as drought, cold, heat, salinity, and oxidative stress (Rapala-Kozik et al., 2008; Yu et al., 2014; Meng et al., 2017; Amjad et al., 2021; Qian et al., 2022). Thiamine can improve the oxidation state of mitochondria, enhance the activities of pyruvate dehydrogenase (PDH), release ROS quickly when stimulated by stress, and activate downstream genes to induce stress resistance in plants. Thiamine thiazole synthase (THI1), also known as 4-methyl-5-hydroxyethyl thiazole phosphate (HET-P) synthase, is a major enzyme in thiamine biosynthesis, and it performs a crucial function in controlling thiamine production in plants (Chen et al., 2022). Nodule expansion and seed development in *Lotus japonicus* depend on the thiamine synthesis controlled by *THI1* (Nagae et al., 2016). Concurrently, *THI1* reacted to various abiotic stimuli, which may improve mitochondrial DNA damage tolerance (Rapala-Kozik et al., 2012). The expression of *THI* was upregulated in *Elaeis guineensis* (oil palm) under osmotic, salinity, and oxidative stress, and was also found to be predominantly upregulated in the early phase (2–6 h) of the NaCl or sorbitol treatment in *Arabidopsis* (Rapala-Kozik et al., 2012; Abidin et al., 2016). When temperatures increased, *THI1* was more prevalent than usual, while thiamine concentrations were lower than usual in *Oryza meridionalis* (Scafaro et al., 2010). *THI1* plays a vital role in the drought responsiveness in *Arabidopsis* and the abscisic acid (ABA) signaling in guard cells (Livak and Schmittgen, 2000). To regulate ABA activation of slow-type anion channels and ABA-induced stomatal closure in *Arabidopsis, THI1* can inhibit Ca^2+^-dependent protein kinase (CPK33) activity in a plasma membrane-restricted way (Li et al., 2016).

A significant amount of *Medicago sativa*, also known as alfalfa, is grown globally as a leguminous feed crop. Alfalfa is essential for the growth of herbivorous animal husbandry and agricultural sustainability due to its high protein content and palatability and symbiotic nitrogen fixation (Yang et al., 2021). The alfalfa acreage has significantly risen in China since the Chinese government has started a strategy to strengthen the milk and alfalfa sectors in 2012 (Fan et al., 2018). Although the deep root structure of alfalfa protects it from drought on the dry and semi-arid ground, the scanty and erratic precipitation of the region has severely hampered crop yields and quality, stifling the growth in the alfalfa business (Zhang and Shi, 2018; Zhao et al., 2019). Enhancing drought resistance of alfalfa requires first learning about its molecular process by which the plant reacts to drought stress. Even though numerous *THI1* genes have important activities in other plants, little is known about *THI1* in alfalfa. In this investigation, the *MsTHI1* was extracted from alfalfa and was characterized. The transcription levels of *MsTHI1* in reaction to cold, drought, salt, and alkalinity were tested to identify how *MsTHI1* is expressed in response to abiotic stresses. The physiological functions of *MsTHI1* under drought stress have also been studied by overexpressing *MsTHI1* in *Nicotiana benthamiana* (tobacco) and alfalfa. In addition, the regulation mechanism was illustrated by quantifying the expression of associated genes involved in the thiamine pathway and stress response. Our findings demonstrated that *MsTHI1* upregulation increased drought resistance by boosting photosynthetic activities, decreasing ROS formation, and preserving osmotic stability. This study will improve our knowledge on how alfalfa reacts to droughts, which could have implications for stress-resistance breeding efforts.

## Materials and methods

### Plant materials and stress treatments

Alfalfa (*Medicago sativa* L. cv. Longmu 801) seeds were treated with 75% ethanol solution for 30 s, followed by treatment with 10% NaClO solution for 10 min. The seeds were then germinated on moist filter paper in Petri dishes after being cleaned 4–5 times with ultrapure water. When the seedlings were 5 days old, they were transplanted into a pot with nutrient soil, perlite, and vermiculite (1:1:1). They were placed in a growth chamber with 60% humidity and a 16 h photoperiod at 24°C and irrigated with 1/2 Hoagland nutrient solution every 2 days. The seedlings were exposed to stress treatments for 4 weeks at 0 (control), 3, 6, 12, 24, and 48 h. To simulate the effects of drought, salt, and alkaline stress, the alfalfa plants were transplanted into nutritional solutions containing 15% PEG-6000, 150 mM NaCl, and 150 mM NaHCO_3_, respectively. To carry out the cold treatment, the plants were placed in a growth chamber maintained at 4°C. Leaves, stems, and roots were promptly removed, frozen in liquid nitrogen, and stored at −80°C for subsequent examination. The experiment was performed in triplicate.

### Isolation and sequence analysis of *MsTHI1* gene

The total RNA from alfalfa leaves was isolated using an Ultrapure RNA kit (CWBIO, Beijing, China). Following the manufacturer's instructions, first-strand complementary DNA (cDNA) was generated using the HiScript II 1^st^ Strand cDNA Synthesis Kit (+gDNA wiper) (Vazyme, Nanjing, China). The coding DNA sequence (CDS) of *MsTHI1* was obtained *via* PCR with degenerate primers (*MsTHI1*-F/*MsTHI1*-R, Supplementary Table S1) that were designed based on the *MtTHI1* sequence of *Medicago truncatula* retrieved from the National Center for Biotechnology Information (NCBI). The coding uses a 2×Unique^TM^ Taq Master Mix (with Dye) (Novogene, Beijing, China) following the manufacturer's instructions. The PCR product was inserted into the pEASY-Blunt Simple Cloning vector using ClonExpress^®^II One Step Cloning Kit (Vazyme, Nanjing, China) and sequenced by SangonBiotech Co. (Shanghai, China).

DNAMAN software performed multiple comparison determinations (version 9, LynnonBiosoft). The phylogenetic tree was generated using MEGA X software and the neighboring connection method. MEME was employed to examine the conserved motif (http://memesuite.org/). Protparam (http://expasy.org/Proteomics) was used to explore the physical and chemical characteristics of polygenes. The secondary structure of the protein was anticipated by the web application, SPOMA (http://pbil.ibcp.fr). The 3D structure of the *MsTHI1* amino acid sequence was predicted using SWISS-MODEL (https://swissmodel.expasy.org/). Using CELLOv.2.5 (http://cello.life.nctu.edu.tw/), the subcellular localization of amino acid sequences in alfalfa was predicted.

### Subcellular localization of *MsTHI1* protein

The coding region of *MsTHI1* without a stop codon was amplified using specific primers (*MsTHI1*-BamHI-F/*MsTHI1*-SacI-R, Supplementary Table S1) with BamHI and SacI restriction sites. The amplicons were double-digested with SacI and BamHI, followed by joining with pCAMBIA-1300 to create a fusion plasmid, pCAMBIA1300-MsTHI1-green fluorescent protein (GFP). The *MsTHI1* fusion protein was transformed into the epidermal cells of *N. benthamiana* through the infiltration of *Agrobacterium tumefaciens*. A confocal laser scanning microscope was used to identify the fluorescence signal after 48 h incubation at 25°C in the dark (Leica TCS SP2 AOBS, Germany).

### Quantitative real-time PCR analysis

Quantitative real-time PCR (qPCR) was used to determine how *MsTHI1* is expressed in different tissues and how it responds to environmental stress in transgenic alfalfa. Total RNA was removed from each piece of harvested tissue and reverse transcribed into cDNA. The CDS of *MsTHI1* was used to make the qPCR primers for *MsTHI1*. The GAPDH gene was utilized in alfalfa as an internal control (He et al., 2020). In transgenic tobacco plants, the tobacco *actin* gene (*NtActin*) was used as a reference gene. Using the online NCBI Primer-BLAST (http://www.ncbi.nlm.nih.gov/tools/primer-blast/), gene-specific primers for thiamine pathway genes were made and are listed in Supplementary Table S1. Three separate biological replicates and three identical reactions were done with each sample. The 2^−Δ*ΔCT*^ comparative method was used to determine how each gene was expressed (Livak and Schmittgen, 2000).

### Plant transformation and generation of transgenic plants

For generating transgenic tobacco plants, the gene-specific primers (*MsTHI1*-PstI-F/*MsTHI1*-BamHI-R, Supplementary Table S1) of *MsTHI1* were inserted into the pCAMBIA1300 vector to make pCAMBIA1300-MsTHI1 expression vector. Afterward, the recombinant vector was put into the *A. tumefaciens* strain, EHA105. Then, the recombinant vector was introduced into EHA105, and transformed into wild-type (WT) tobacco by the agroinfiltration method (Kaur et al., 2021). A 20 mg·L^−1^ hygromycin (Hyg) was put into the medium for seed germination to find transgenic lines (T1) that are resistant to Hyg. Utilizing specific primers (*Hyg*-F/*Hyg*-R, Supplementary Table S1), a PCR test was used to detect the *Hyg* gene in homozygous transformants (T2), which was validated by qPCR using specific primers for *MsTHI1*. *NtActin* was used as a control within the lab. For more drought stress tests, lines 2 (OV#Nt2), 3 (OV#Nt3), and 7 (OV#Nt7) from T2-generation homozygous lines and WT lines were utilized.

To create transgenic alfalfa plants, the gene-specific primers (*MsTHI1*-SpeI-F/*MsTHI1*-XbaI-R, Supplementary Table S1) of *MsTHI1* were inserted into the pMDC123 vector to make pMDC123-MsTHI1 expression vector. After that, the recombinant vector was put into *A. tumefaciens* strain, LB4404, and transformed into the cotyledon nodes of alfalfa using the agroinfiltration method (Sun et al., 2020). Since the pMDC123 vector has a *Bar* resistance gene that makes it resistant to the herbicide glufosinate, 1.0 mg·L^−1^ of glufosinate-ammonium was used to pick out the transformants. The shoots grown back were planted in 1/2 Murashige and Skoog (MS) medium. The herbicide-resistant plants were then put through a PCR test to find the *Bar* gene by employing specific primers (*Bar*-F/*Bar*-R, Supplementary Table S1), which was affirmed by qPCR using specific primers for *MsTHI1*. The *GAPDH* gene from alfalfa was used as a control for the study. The homozygous lines were then passed on and used in more tests to find how drought affects plants.

### Drought tolerance assay

On 1/2-strength MS medium plates, seeds of WT tobacco and transgenic tobacco lines were planted and kept at 4°C for 2 days before being moved to a growth chamber at 25°C for 7 days. After the seeds were germinated, they were moved to a greenhouse and put in plastic containers with vermiculite. Drought resistance tests were done on 3-week-old plants. The WT alfalfa and transgenic alfalfa were spread by cutting. Then, the same WT and transgenic plants were moved into plastic pots with vermiculite and put in a greenhouse. Drought tolerance tests were done on 6-week-old plants. For drought tolerance tests, the plants were irrigated for a week with a nutrient solution with 15% PEG-6000. Afterward, the treated leaves of the plants were picked right away, frozen in liquid nitrogen, and kept at −80°C for subsequent use.

### Measurement of physiological and biochemical changes

Stress-treated WT, transgenic plants, and three categories of biological repeats, in addition to the three technical repeats, were set up at each time point. A kit was used to quantify the amount of VB1 based on the manufacturer's recommendations (Comin, Suzhou, China). The accumulation of malondialdehyde (MDA) was determined by a modified thiobarbituric acid (TBA) method as described by Puckette et al. (2007). The relative chlorophyll content [soil and plant analyzer development (SPAD) value] was measured by the SPAD-502 Chlorophyll Meter, and three leaves from each group were used to determine the value. Three measurements were made to find the average SPAD value. Three leaves kept in the dark for 20 min were measured thrice to get an average Fv/Fm value. The amount of superoxide anion radical (${\text{O}}_{2}^{.-}$) was measured by making some changes to the procedure reported by Jiang and Zhang (2001). The nitroblue tetrazolium (NBT) method (Giannopolitis and Ries, 1972) with a few minor changes were used to test the activity of superoxide dismutase (SOD, EC1.15.1.1). The guaiacol method was used to measure the peroxidase activity (POD, EC 1.11.1.7) (Polle et al., 1994). The amount of proline (Pro) was found using spectrophotometry and the ninhydrin method (Zhao et al., 2009). Wellburn and Lichtenthaler (1984) found out the quantity of chlorophyll *a* (Chl *a*) and chlorophyll *b* (Chl *b*) present in plants. Tietze (1969) used fluorometry to identify reduced glutathione (GSH) in the body. The Bradford method was employed to determine the soluble protein (Bradford, 1976).

### Expression analysis of related genes

In WT and transgenic alfalfa, the expression of eight genes associated with the thiamine pathway and stress response was analyzed by employing qPCR; this was done to show how *MsTHI1* helps plants deal with drought. Some of these genes, like thiazole biosynthetic enzyme (THIAMIN4, *THI4*), 4-amino-2-methyl-5-hydroxymethyl pyrimidine phosphate (HMP-P) kinase/thiamin monophosphate (TMP) pyrophosphorylase (*TH1*), thiamine pyrophosphokinase (*TPK1*), chaperonin-like RbcX protein 2 (*RbcX2*), SOD [Cu-Zn] (*Cu/Zn-SOD*), and calcium-dependent protein kinases (*CPK13, CPK17*, and *CPK32*) play critical roles in thiamine synthesis and abiotic stress response. The *GAPDH* gene was utilized as an internal reference gene, and Supplementary Table S1 lists the primers employed in this study.

### Data analysis

All experiments presented in this study were done three times in the same way. For statistical analysis with SPSS 22.0, the Student's *t*-test and one-way analysis of variance were used to identify significant differences.

## Results

### Isolation and characteristics of *MsTHI1*

The *MsTHI1* CDS (GenBank accession: MH206189) encoded a protein of 350 amino acids with three putative thiazole synthesis domains, 20 essential amino acids, 28 positively charged amino acid residues (Arg, Lys), and 36 negatively charged amino acid residues (Asp, Glu) (Supplementary Figure S1A). The *MsTHI1* has the chemical formula of C_1623_H_2605_N_441_O_500_S_19_, the molecular weight (MW) of 36.91 kDa, and a predicted isoelectric point (PI) of 5.68. *MsTHI1* protein aqueous solution at 280 nm has an extinction coefficient of 18,575, fat coefficient of 90.3, an average hydrophilic coefficient of 0.112, and an instability coefficient of 33.24, suggesting the stability of the protein. The neighbor-joining approach used to generate the phylogenetic tree showed that *MsTHI1* and MtTHI1 were on the same branch and that *MsTHI1* was the most similar to *MtTHI1* (Supplementary Figure S1B). *MsTHI1* protein, as accurately predicted by its three-dimensional structure, consists of 32.86% α-helices, 18.57% β-extension chain, 8.29% β-rotation angle, and 40.29% irregular curls (Supplementary Figure S1C).

### Subcellular localization of *MsTHI1*

**Figure 1:**
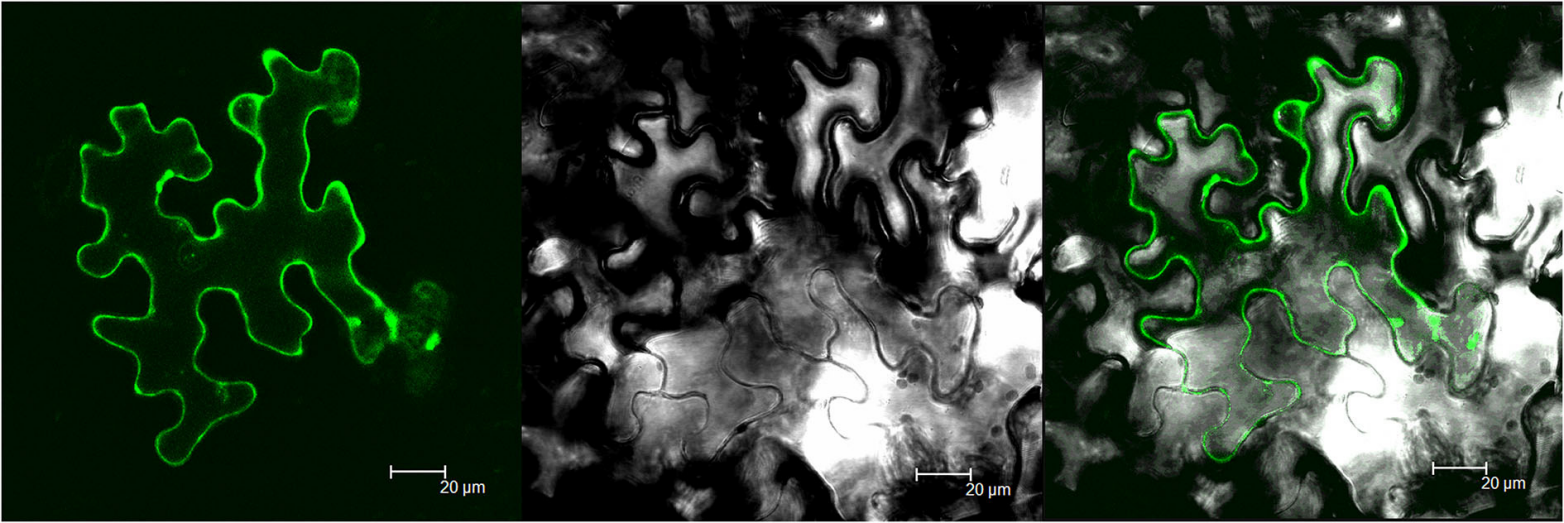
Subcellular localization of *MsTHI1*. **(A)** fusion GFP fluorescence. **(B)** bright field under infected leaves, **(C)** fusion picture. Scale bar, 20 μm.

To investigate the subcellular distribution of *MsTHI1*, a GFP-MsTHI1 fusion protein was transiently coexpressed in *N. benthamiana* leaves and then visualized using laser scanning confocal microscopy. MsTHI1-GFP fluorescence was found in the cell membrane and chloroplasts, as evidenced by the brilliant field under infected leaves, as well as the fusion picture (Figure 1). However, predictions of CELLO 2.5 to find where *MsTHI1* is subcellularly localized suggested that it is most likely to be found in chloroplasts (4.275). Therefore, the *MsTHI1* protein was primarily found in chloroplasts.

### *MsTHI1* expression patterns

**Figure 2:**
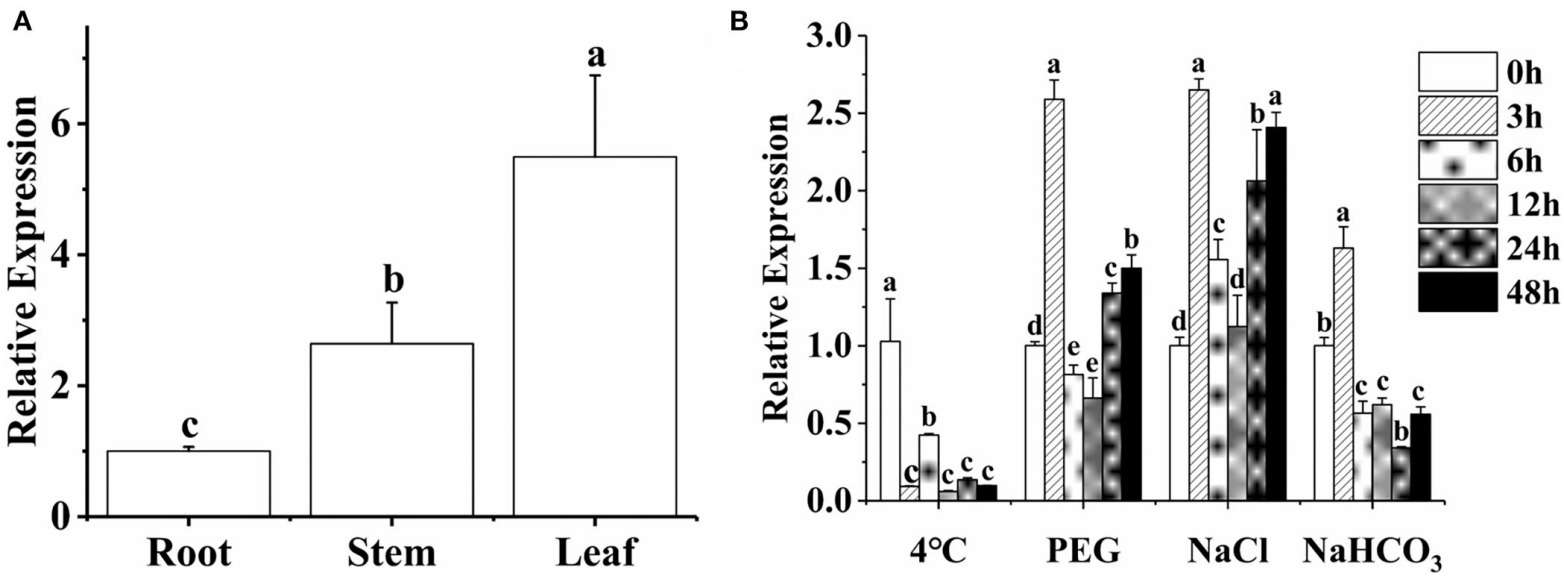
Expression pattern of *MsTHI1*. **(A)** Tissue-specific expression of *MsTHI* in the alfalfa root, stem, and leaf. **(B)** Expression analysis of *MsTHI1* in leaves of alfalfa subjected to cold (4°C), drought (15% PEG), salt (150 mM of NaCl), and alkaline (150 mM of NaHCO_3_) stresses. The expression levels were normalized in the alfalfa *GAPDH* gene. Mean ± standard deviations from three biological replicates are shown with error bars. These letters denote variations that are statistically significant (α = 0.05).

The expression profiles of *MsTHI1* in distinct alfalfa tissues and its sensitivities to drought, cold, salt, and alkaline stress were studied through qPCR. The findings demonstrated that the expression pattern of *MsTHI1* in alfalfa leaves was more significant than in the roots and stems (Figure 2A). The expression level of *MsTHI1* was significantly suppressed during cold stress (Figure 2B). The expression level of *MsTHI1* changed comparably with 15% of PEG and 150 mM of NaCl treatment. They peaked at 3 h, dropped at 6 and 12 h, and climbed once more at 24 and 48 h. Contrary to the *MsTHI1* upregulation under salt stress, the *MsTHI1* expression was significantly suppressed at 6 and 12 h under drought stress. The expression level of *MsTHI1* at 150 mM of NaHCO_3_ peaked at 3 h, followed by a drop from 6 to 48 h. These findings indicated that *MsTHI1* expression was tissue-specific in alfalfa and that *MsTHI1* might implicate the self-protection mechanisms of alfalfa in response to aggressive ecological scenarios.

### The ability of transgenic plants to tolerate drought is enhanced by *MsTHI1* upregulation

**Figure 3:**
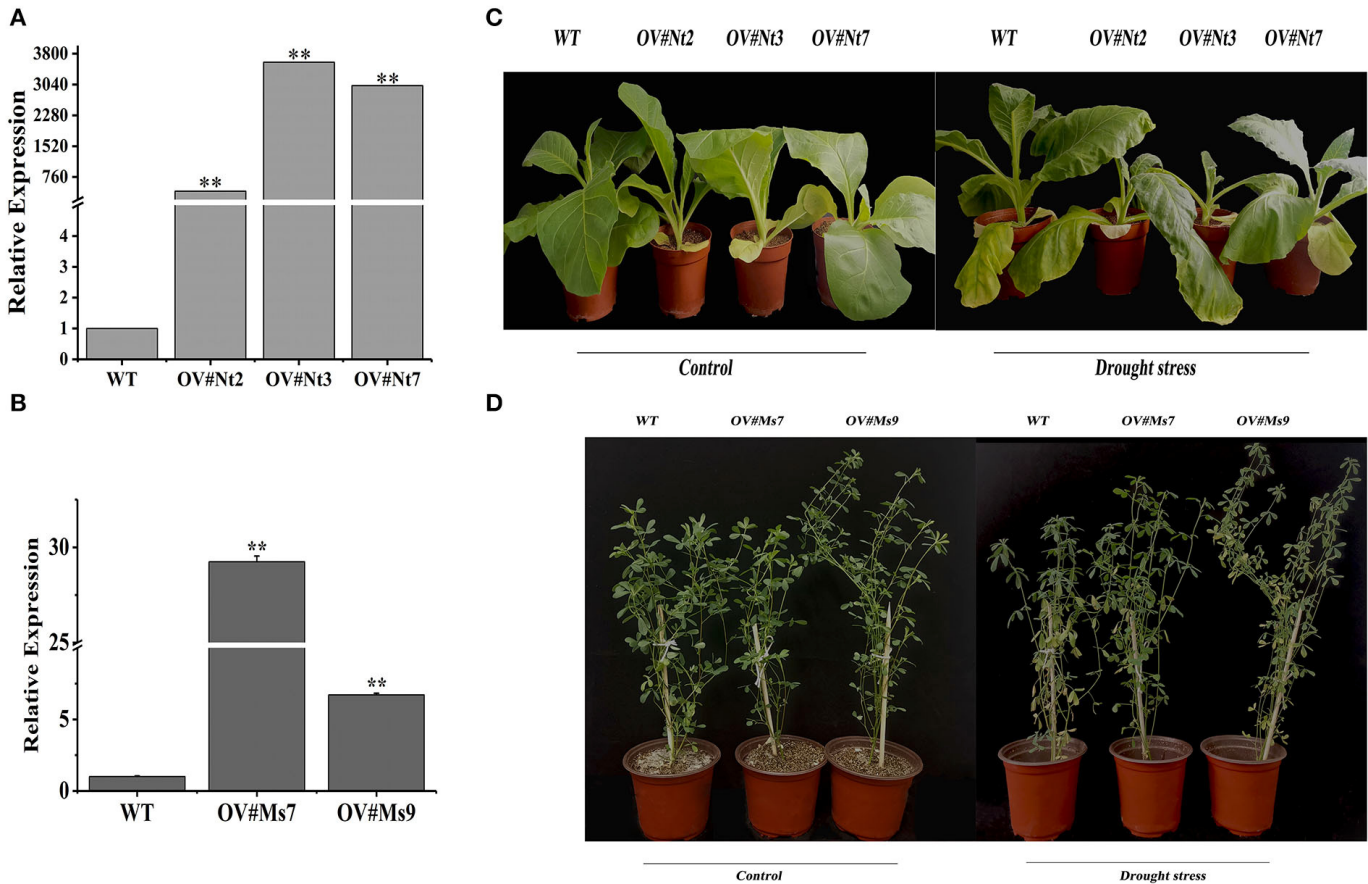
Identification of transformation with *MsTHI1 via* qPCR and phenotype of transformation with *MsTHI1* under control conditions and drought stress. **(A)** Identifying transgenic tobacco *via* quantitative real-time PCR (qPCR). **(B)** Identifying transgenic alfalfa *via* qPCR. **(C)** Phenotype of three-week-old transgenic tobacco plants with overexpressed *MsTHI1* under control and drought stress for seven days. **(D)** Phenotype of six-week-old transgenic alfalfa plants with overexpressed *MsTHI1* under control and drought stress for seven days. *NtActin* and alfalfa *GAPDH* gene were used as the internal reference control for identifying transgenic tobacco and alfalfa, respectively, *via* qPCR. Mean ± standard deviations from three biological replicates are shown with error bars. “**”: extremely significant difference between WT and transformation line (*P* < 0.01). WT: wild type; OV#Nt2, OV#Nt3, and OV#Nt7: transgenic tobacco lines 2, 3, and 7, respectively. OV#Ms7 and OV#Ms9: transgenic alfalfa lines 7 and 9, respectively.

Transgenic tobacco (T2) and alfalfa plants were created and validated with Hyg and *Bar*, respectively, through PCR to examine the involvement of *MsTHI1* in the plant drought tolerance (Supplementary Figure S2). *MsTHI1* transcript levels in OV#Nt2, OV#Nt3, as well as OV#Nt7 transgenic lines were 413.6, 3593.0, and 3014.8 times greater than those in WT tobacco plants, accordingly (Figure 3A). The expression of *MsTHI1* was 29.2 and 6.9 times greater in OV#Ms7 and OV#Ms9 transgenic lines, respectively, compared to that in WT alfalfa plants (Figure 3B). No phenotype was observed between *MsTHI1*-transformed and WT plants under normal control conditions in either transgenic tobacco or transgenic alfalfa (Figures 3C,D). When exposed to dryness for a week, the leaves of both WT tobacco and alfalfa plants wilted, and the growth was significantly suppressed. Despite this, OV#Nt2, OV#Nt3, OV#Nt7, OV#Ms7, and OV#Ms9 continued to develop properly with only a few yellow leaves. The results demonstrated that the drought resistance of transgenic plants was enhanced by overexpressing *MsTHI1*.

### Modifications in the physiology of transgenic plants that overexpress *MsTHI1* during conditions of drought stress

**Figure 4:**
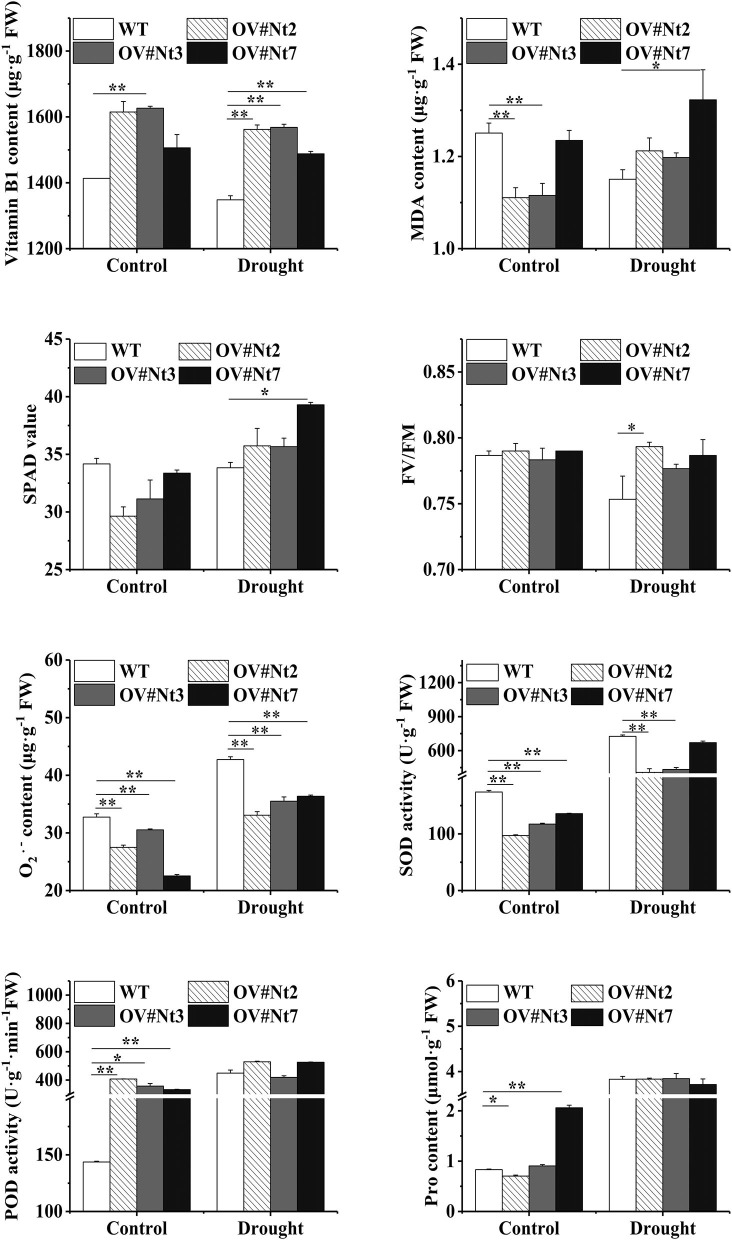
Modifications in the physiology of transgenic tobacco plants that overexpress *MsTHI1* during control or under drought stress. Mean ± standard deviations from three biological replicates are displayed with error bars. MDA, malondialdehyde. SPAD, soil and plant analyzer development. Fv/Fm, the PS II primary light energy conversion efficiency. ${\text{O}}_{2}^{.-}$, superoxide anion radical. SOD, superoxide dismutase. POD, peroxidase. Pro, proline. WT, wild type. OV#Nt2, OV#Nt3, and OV#Nt7, transgenic tobacco lines 2, 3, and 7, respectively. “*”, significant difference between WT and transgenic tobacco lines (*P* < 0.05). “**”, extremely significant difference between WT and transgenic tobacco lines (*P* < 0.01).

To better demonstrate the role of *MsTHI1* in stress conditions tolerance, the physiological modifications in the transgenic tobacco or even alfalfa plants experiencing environmental stresses were studied. Transgenic tobacco with an increased VB1 concentration was found both in normal and drought conditions due to *MsTHI1* upregulation (Figure 4). During normal conditions, MDA levels in WT plants were higher than in OV#Nt2 and OV#Nt3 species. Even though the SPAD value of the transgenic tobacco was lesser than that of the WT plants in the control environment, all plants increased their SPAD value after being subjected to drought treatment, with OV#Nt7 plants showing the most significant increase. During drought conditions, the Fv/Fm ratio decreased more quickly in WT plants than in transgenic tobacco, with OV#Nt2 plants having the highest Fv/Fm ratio (0.79). Following drought stress, the WT and transgenic tobacco showed an increase in ${\text{O}}_{2}^{.-}$ concentration and SOD and POD activities. While WT plants increased their ${\text{O}}_{2}^{.-}$ level quicker than the transgenic tobacco, the latter eventually caught up. Considering drought stress, there was no significant difference between the WT and transgenic tobacco in free Pro concentration.

During drought conditions, VB1 levels in transgenic and WT alfalfa decreased, but the transgenic plants maintained a greater VB1 concentration than the WT ones. During drought conditions, MDA levels of transgenic and WT alfalfa were elevated, whereas the MDA level of transgenic alfalfa was lower under control or drought conditions. Chl *a* and Chl *b* concentrations were reduced during drought and were increased in *MsTHI*-transformed plants, mirroring the pattern of VB1. Transgenic and WT alfalfa both elevated their ${\text{O}}_{2}^{.-}$ levels in response to drought conditions, whereas the ${\text{O}}_{2}^{.-}$ level of transgenic alfalfa remained lower in control and drought conditions. However, the functions of SOD and the concentrations of GSH in transgenic alfalfa plants were significantly decreased compared to WT plants during control and droughts scenarios, confirming the role of VB1 in alfalfa drought tolerance. Transgenic alfalfa and WT plants exhibited statistically significant elevations in POD activity and Pro content in response to drought conditions.

### Expression analysis of associated genes in the overexpression of *MsTHI1* in transgenic alfalfa under drought stress

Eight associated genes involving *THI4, TH1, TPK1, RbcX2, Cu/Zn-SOD, CPK13, CPK17*, and *CPK32* were assessed for their expression patterns in transgenic and WT alfalfa plants under drought conditions using qPCR. As depicted in **Figure 6**, drought stress dramatically upregulated the expression of *THI4, TPK1, RbcX2*, and *CPK32* in transgenic and WT alfalfa plants. Intriguingly, during drought stress, *TH1* and *CPK17* expression levels were reduced in transgenic alfalfa while they were increased in WT alfalfa. During drought conditions, *Cu/Zn-SOD* expression did not alter much in the transgenic alfalfa, whereas it was significantly upregulated in WT alfalfa. During drought stress, the *CPK13* expression was elevated in the transgenic alfalfa but suppressed in WT plants.

## Discussion

To support proper metabolic activity, people depend on an adequate thiamin intake from food. Metabolic engineering to boost thiamin production in plants is a promising approach to reducing VB1 deficiency and, by extension, improving worldwide human health (Strobbe et al., 2021). In addition to its established roles in thiamine synthesis and mitochondrial DNA damage resistance, *THI1* contributes significantly to plant abiotic response to stress (Rapala-Kozik et al., 2012; Li et al., 2016). Several plant *THI1* genes that respond to drought, salinity, heat, and oxidative stress have been identified (Livak and Schmittgen, 2000; Scafaro et al., 2010; Abidin et al., 2016; Mangel et al., 2017; Chen et al., 2022), but no research has been conducted on alfalfa. Here, we report the isolation and functional characterization of a *THI1* gene (called *MsTHI1*) from alfalfa. The protein encoded by *MsTHI1* is 350 amino acids long. It has three putative thiazole synthesis domains (Supplementary Figure S1A), all of which bind to 2-carboxylate-4-methyl-5-beta-(ethyladenosine 5'-diphosphate) thiazole, a possible intermediate in the thiazole biosynthesis of Eukaryotes (Godoi et al., 2006). The MsTHI1 protein was localized mainly in the chloroplast of plant cells (Figure 1), as is the case for the translation initiation at the first AUG (start codons) directs translocation of *THI1* to chloroplasts, indicating a high requirement for *THI1* in chloroplasts (Chabregas et al., 2003). Similar to *THI1* expression in *Arabidopsis* (Teixeira et al., 2005; Dong et al., 2015), the expression level of *MsTHI1* in alfalfa leaves was higher than that in the roots and stems (Figure 2A), confirming the tissue-specific expression of *THI1*. The *MsTHI1* was quickly upregulated in alfalfa leaves within 3 h during drought, salt, and alkaline stress, and downregulated under cold stress (Figure 2B), which might contribute to the self-protection mechanisms of alfalfa in response to unfavorable ecological scenarios. Although the expression of *MsTHI1* was similar in response to drought and salt, *MsTHI1* responses were more sensitive to salt, which may be associated with the characters of the alfalfa cultivar used in the study.

Overexpressing *MsTHI1* can increase drought resistance in transgenic plants. Drought stress significantly stunted the development of WT tobacco and alfalfa plants (Figure 3). To better understand the role of *MsTHI1* in plants during drought conditions, we studied the physiological and biochemical responses of transgenic plants. VB1 is a vital member of the vitamin B group, and the elevation in the level of VB1 is important in alleviating stressful conditions (Amjad et al., 2021). Our results demonstrated that VB1 levels in transgenic tobacco and alfalfa were always significantly higher than in WT plants. However, VB1 contents diminished in WT and transgenic plants under drought conditions suggesting that *MsTHI1* overexpression can counteract drought stress by consuming VB1. The MDA amount was also analyzed as a vital sign of lipid peroxidation in the membrane, reflecting stress-induced molecular damage. Following exposure to drought, transgenic *SbWRKY30 Arabidopsis*, as well as rice plants, had lower levels of MDA than WT plants (Yang et al., 2020). In this case, MDA levels in *MsTHI1*-overexpressed alfalfa were considerably lower than in WT alfalfa during control and drought stress, demonstrating a robust ability to reduce membrane damage. However, the MDA content in the transgenic tobacco was higher than that in the WT tobacco after drought stress. The results may reflect the difference in ROS scavenging capacity and plasma membrane damage in alfalfa and tobacco. Balanced photosynthesis under drought is necessary for better survivability and agricultural benefits in terms of biomass and output (Daszkowska-Golec et al., 2019). Leaf SPAD readings were directly correlated with grain output, and most of the high SPAD genotypes sustained hot canopies under drought (Raina et al., 2019). The chlorophyll contents and Fv/Fm, utilized as reliable benchmark indicators in identifying drought-adapted genotypes, were reduced under drought-treated *Zea mays* (maize) seedlings (Chen et al., 2016). In this study, the SPAD value in OV#Nt7 was substantially higher than that in the WT tobacco under drought stress. Simultaneously, the decline rate of Fv/Fm in the WT tobacco was faster than in transgenic plants during drought conditions. The levels of Chl *a* and Chl *b* reduced during drought conditions, but the alfalfa transformed with *MsTHI* had more significant Chl *a* and Chl *b* levels. These results revealed that *MsTHI1* upregulation might have a function of controlling chlorophyll content to balance photosynthesis, thus conferring a degree of drought conditions.

**Figure 5:**
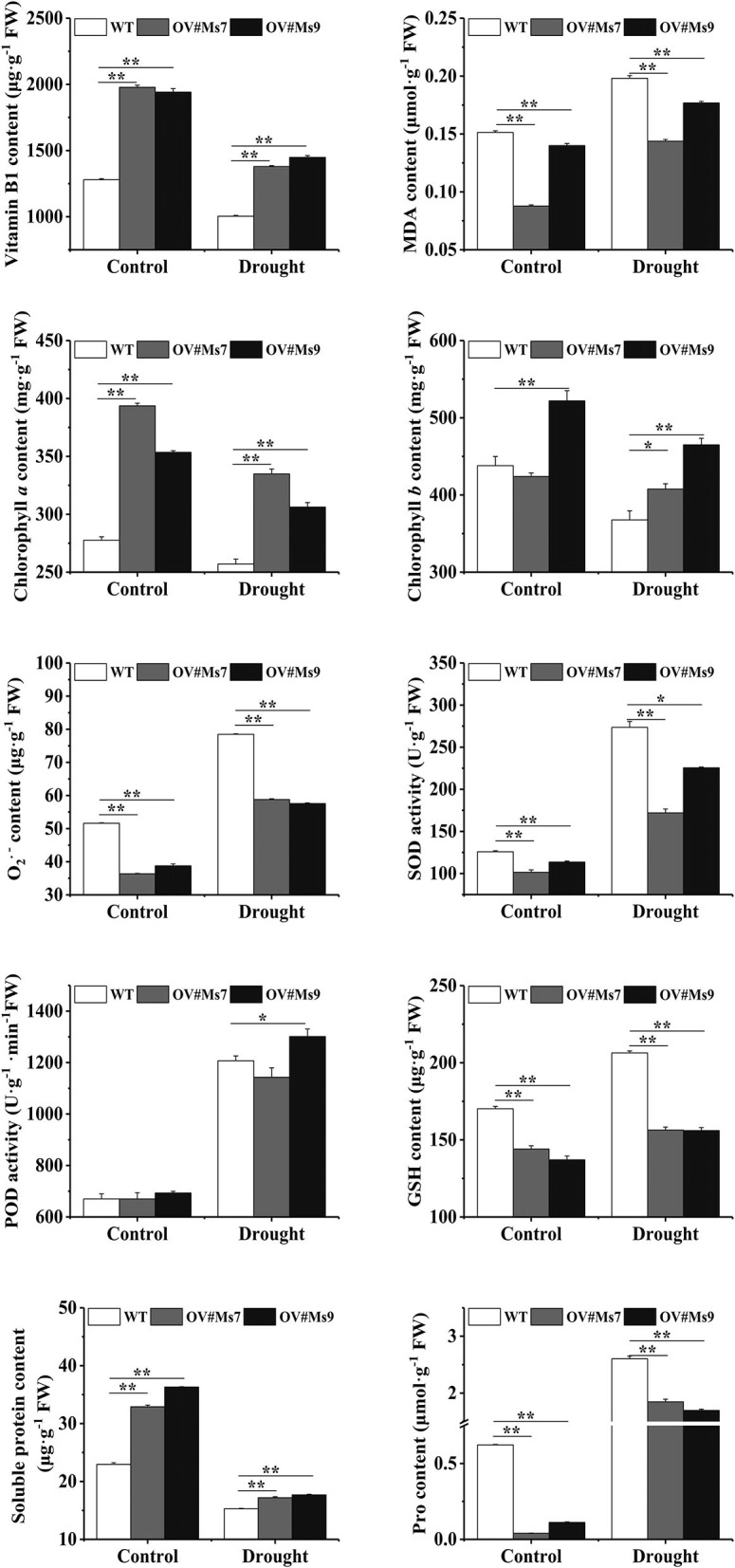
Physiological changes of transgenic alfalfa plants with overexpressing *MsTHI1* under control and drought stress. Mean ± standard deviations from three biological replicates are depicted with error bars. MDA, malondialdehyde. ${\text{O}}_{2}^{.-}$ superoxide anion radical. SOD, superoxide dismutase. POD, peroxidase. GSH, reduced glutathione. Pro, proline. WT, wild type. OV#Ms7 and OV#Ms9, transgenic alfalfa lines 7 and 9, respectively. “*”, significant difference between WT and transgenic tobacco lines (*P* < 0.05). “**”, extremely significant difference between WT and transgenic tobacco lines (*P* < 0.01).

Drought stress can produce an immediate generation of ROS. Plants might elicit an antioxidant protective mechanism to scavenge superfluous ROS and protect the cells against oxidative stress during stressful circumstances (Sharma et al., 2012). The antioxidant enzymes, including SOD and POD, and non-enzymatic antioxidants, including GSH, are critical in detoxifying stress-induced ROS. Herein, we noticed that the transgenic tobacco and alfalfa revealed a lower ${\text{O}}_{2}^{.-}$ level than WT plants during drought conditions (Figures 4, 5). Nevertheless, SOD activities and GSH concentration in the transgenic alfalfa were similarly lesser than in WT plants. The variations in POD activities depend on the expression pattern of *MsTHI1* in transformants. There was no difference in the POD activity between transgenic and WT tobacco under drought stress. Conversely, the concentration of soluble protein fell quickly during drought conditions. Previous findings have indicated that the rise of VB1 by stimulating thiamine metabolism protected cell organelles and decreased the ROS levels in the cells of *Taxus Chinensis*, and chlorophyll could be employed as an antioxidant (Meng et al., 2017; Agathokleous et al., 2020). Consequently, ROS scavenging in transgenic plants might correlate with *MsTHI1* upregulation, which triggered the thiamine metabolism and the aggregation of chlorophyll to decrease the amount of ROS. The soluble protein and free Pro are key osmotic regulators, which can raise the osmotic potential of cells and boost plant tolerance to stressful conditions (Hosseinifard et al., 2022). In the present research, the soluble protein level in transgenic alfalfa was mainly greater than that in WT alfalfa during control or drought settings. Although the level of Pro in transgenic alfalfa was consistently lower than that in WT alfalfa throughout control and drought situations, there was no difference in the level of Pro between transgenic and WT tobacco under drought conditions. These findings demonstrated that enhancing drought tolerance in transgenic plants might link with the buildup of soluble protein during drought environments.

**Figure 6:**
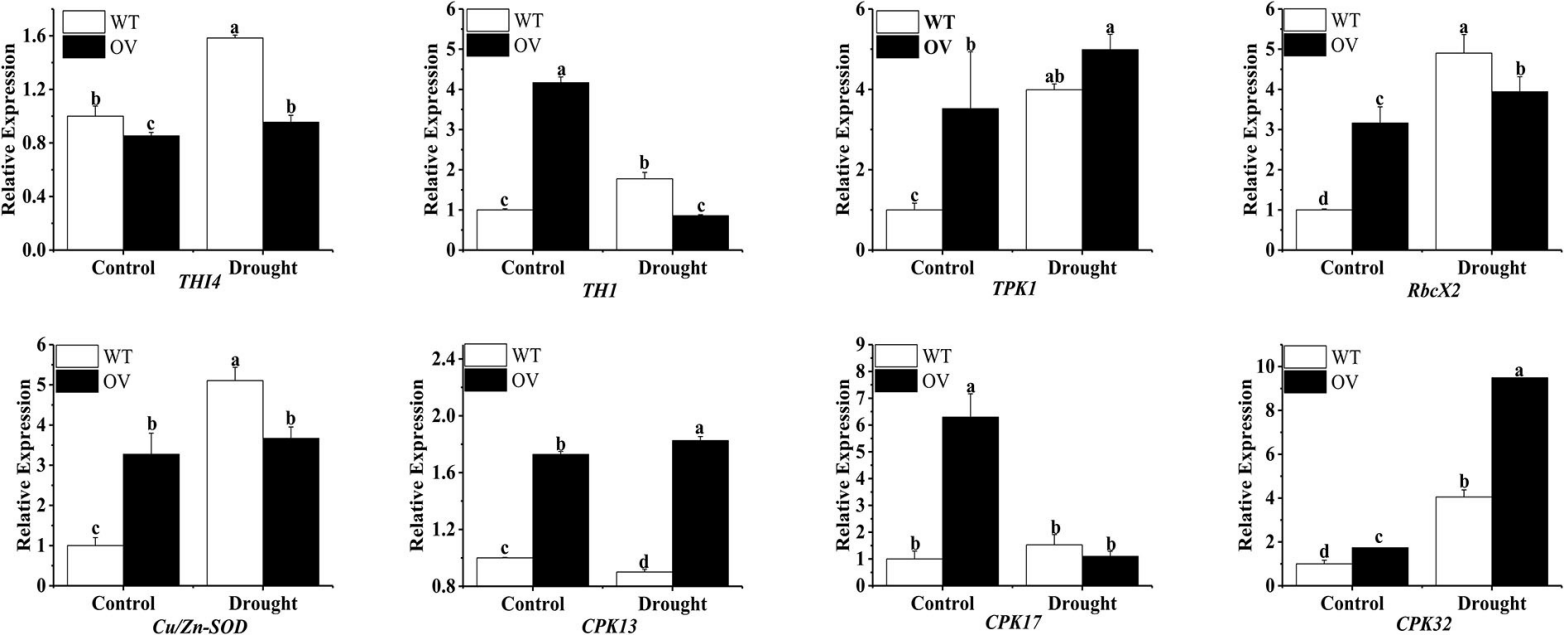
Expression of eight associated genes in transgenic alfalfa plants with overexpressing *MsTHI1* under drought stress. WT, wild-type alfalfa. OV, overexpressing *MsTHI1* alfalfa. *THI4*, thiazole biosynthetic enzyme THIAMIN4. *TH1*, HMP-P kinase/TMP pyrophosphorylase. *TPK1*, thiamine pyrophosphokinase. *RbcX2*, chaperonin-like RbcX protein 2. *Cu/Zn-SOD*, superoxide dismutase [Cu-Zn]. *CPK13, CPK17*, and *CPK32*: calcium-dependent protein kinases. Mean ± standard deviations from three biological replicates are shown with error bars. These letters denote statistically significant variations (α = 0.05).

Upregulated *MsTHI1* alfalfa responds to drought conditions by upregulating genes involved in the thiamine system and stress response. The suicide enzyme, THI4 is responsible for mediating the production of the thiazole precursor of thiamin (cThz-P), and hence, it must be destroyed and resynthesized (Sun et al., 2019). *Verticillium dahliae*, a disease of vascular plants, is more resistant to stresses like UV damage and oxidative stress due to *VdTHI4* (Hoppenau et al., 2014). *TH1*, a thiamine phosphate synthase, was upregulated in response to oxidative stress due to its involvement in the phosphorylation of HMP-P from plant thiamine thiazole to HMP-pyrophosphate (HMP-PP) (Strobbe et al., 2021). TPK is a crucial enzyme in the biosynthetic pathways of thiamine, and during environmental stresses, TPK activities were significantly increased in regulating thiamine metabolism in maize seedlings (Rapala-Kozik et al., 2008). During drought conditions, this research found that *THI4* and *TPK1* transcription levels were elevated in transgenic alfalfa, whereas *TH1* levels were declined (Figure 6). *RbcX2* can carboxylate Rubisco, which boosts photosynthesis and can also fold proteins to control the accumulation of soluble protein and drought resistance in plants (Liu et al., 2010; Doron et al., 2020). One form of SOD, Cu/Zn-SOD, is fundamental due to its role in ROS elimination and its correlation with drought tolerance in plants (Saed-Moucheshi et al., 2021; Zhang et al., 2021). Both transgenic and WT alfalfa showed an increase in *RbcX2* transcription in response to drought stress, while WT alfalfa showed a marked increase in *Cu/Zn-SOD* transcription. Changes in the content of soluble protein and SOD activity in the drought tolerance experiment of transformants were in line with these findings. Serine/threonine protein kinases, of which CPKs are a subfamily, interact with *THI1* to confer resistance to abiotic stressors, such as dehydration, salinity, and low temperature (Atif et al., 2019; Zhang et al., 2020; Chen et al., 2021). Variations in *CPK13, CPK17*, and *CPK32* transcription were observed in the present study. These findings indicated that CPKs might have a role in regulating the response of *MsTHI1* to drought stress; however, the mechanisms by which CPKs regulated *MsTHI1* expression still require investigation.

**Figure 7:**
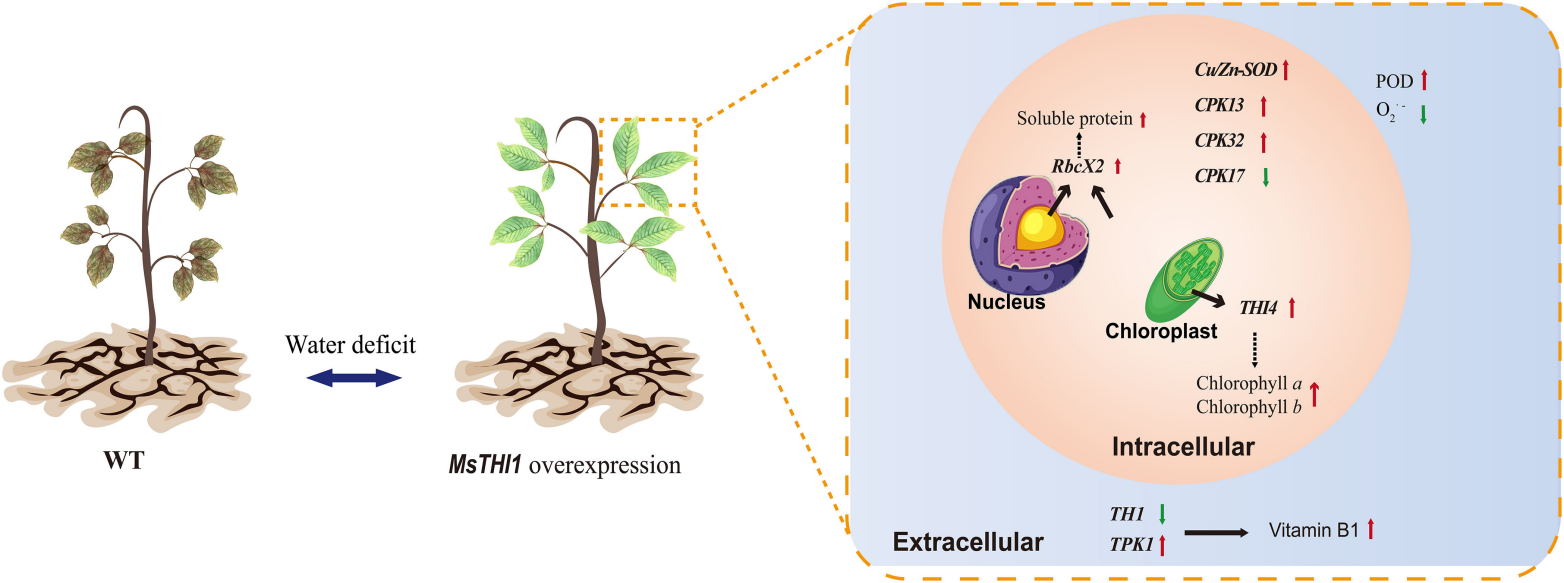
Possible function model of *MsTHI1* response to drought stress. The red arrows represent significant increases in physiological signs or genes being overexpressed. Green arrows represent decreased expressions of genes or declines in physiological markers. Dotted lines represent potential regulating pathways. *CPK13, CPK 17*, and *CPK32*: calcium-dependent protein kinases. *Cu/Zn-SOD*, superoxide dismutase [Cu-Zn]. ${\text{O}}_{2}^{.-}$, superoxide anion radical. POD, peroxidase. *RbcX2*, chaperonin-like RbcX protein 2. *TH1*, HMP-P kinase/TMP pyrophosphorylase. *THI4*, thiazole biosynthetic enzyme THIAMIN4. *TPK1*, thiamine pyrophosphokinase. WT, wild type.

Accordingly, we postulated that *MsTHI1* participation in the plant drought tolerance might include a complicated regulating network that includes the physiological and gene expression levels (Figure 7). The overexpression of *MsTHI1* can enhance the transcription of *THI4, TPK1, RbcX2, Cu/Zn-SOD, CPK13*, and *CPK32*, and decrease the transcription of *TH1* and *CPK17* to increase the accumulation of VB1, Chl *a*, Chl *b*, soluble protein, and POD activities, which helps preserve osmotic balance and strengthens photosynthesis and ROS scavenging.

## Conclusion

Overall, we could identify the thiamine thiazole synthase, *MsTHI1*, from alfalfa and determine its beneficial effect on the enhancement of drought stress tolerance. *MsTHI1* localized to chloroplasts and alfalfa leaves exhibited excellent expression levels. Simultaneously, exposure to stressful conditions increased *MsTHI1*. The upregulation of *MsTHI1* enhanced drought tolerance in plants and led to an enhancement in photosynthesis by increasing SPAD, maintaining Fv/Fm, and aggregating Chl *a* and Chl *b*, reducing the ROS accumulation and lipid peroxidation by elevating VB1 synthesis and POD activity, reducing MDA, and maintaining the balance of osmotic by strengthening the accumulation of soluble protein. Moreover, *MsTHI1* mediated signal transduction by upregulating the transcription of *THI4, TPK1, RbcX2, Cu/Zn-SOD, CPK13*, and *CPK32*, and downregulating the transcription of *TH1* and *CPK17* in transgenic alfalfa under drought stress. Our findings shed light on the mechanism by which *MsTHI1* alleviates plant drought stress and suggest that it should be incorporated into successful breeding initiatives to enhance the tolerance of alfalfa to drought.

## Data availability statement

The datasets presented in this study can be found in online repositories. The names of the repository/repositories and accession number(s) can be found in the article/Supplementary material.

## Author contributions

HY, GC, and PZ designed the experiment. HY experimented and wrote the manuscript. ZW, HL, YZ, and MY performed part of the experiment and analyzed and discussed data analysis. GC and PZ supervised the project and revised the manuscript. All authors agree to be accountable for the content of the work, contributed to the article and approved the submitted version.

## Funding

This research was funded by the Heilongjiang Provincial Natural Science Foundation of China (YQ2021C019).

## Conflict of interest

The authors declare that the research was conducted in the absence of any commercial or financial relationships that could be construed as a potential conflict of interest.

## Publisher's note

All claims expressed in this article are solely those of the authors and do not necessarily represent those of their affiliated organizations, or those of the publisher, the editors and the reviewers. Any product that may be evaluated in this article, or claim that may be made by its manufacturer, is not guaranteed or endorsed by the publisher.

## Supplementary material

Supplementary Figure S1 Alignment of homologous protein sequences. **(A)**. phylogenetic tree generation **(B)**, and three-dimensional modeling of *MsTHI1* **(C)**.

Supplementary Figure S2 Identification of transformation with *MsTHI via* PCR. **(A)**. PCR identification of transgenic tobacco with *Hyg*. **(B)**. PCR identification of transgenic alfalfa with *Bar*. M, Trans2K DNA Marker; “-”, negative control; “+”, positive control; WT, wild type; number, transformation line.

Supplementary Table S1 List of primers used in this study.
