# Supplementary figures and images for: MsTHI1 overexpression improves drought tolerance in transgenic alfalfa (Medicago sativa L.)
Source: Front Plant Sci. 2022 Sep 8;13:992024. doi: 10.3389/fpls.2022.992024 (PMC9495609; doi:10.3389/fpls.2022.992024)

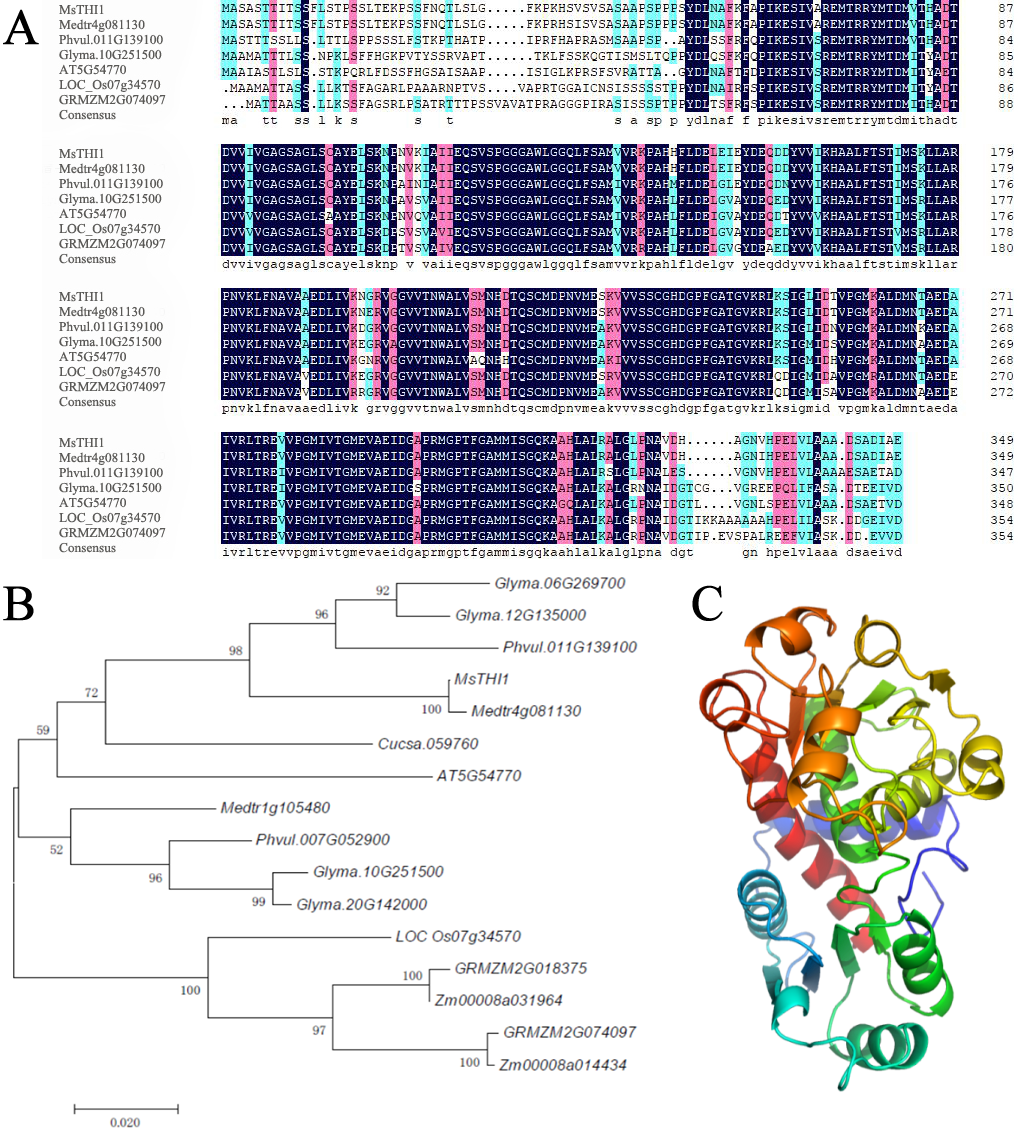

Supplement: Supplementary Figure S1 — Alignment of homologous protein sequences. (A). phylogenetic tree generation (B), and three-dimensional modeling of MsTHI1 (C). [file Image_1.TIF]

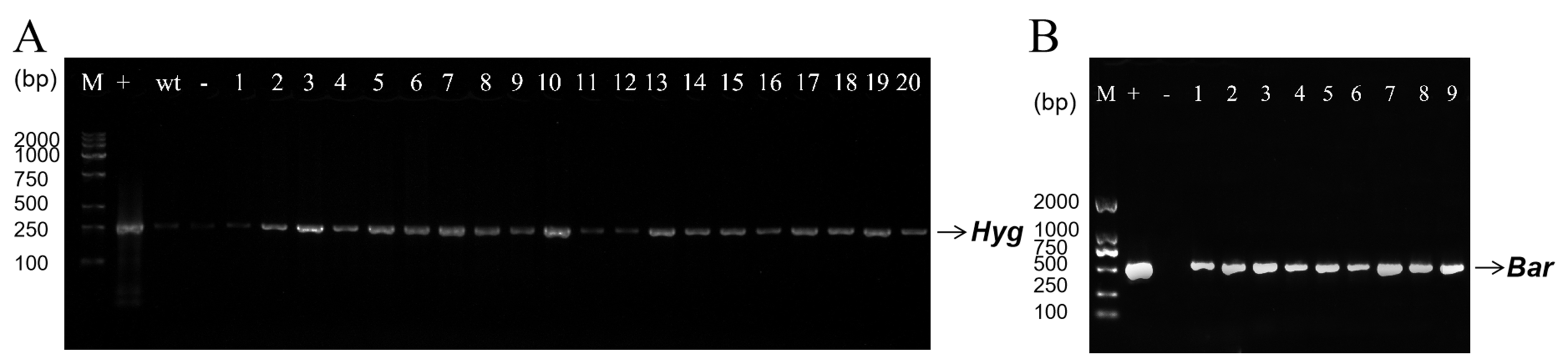

Supplement: Supplementary Figure S2 — Identification of transformation with MsTHI via PCR. (A). PCR identification of transgenic tobacco with Hyg. (B). PCR identification of transgenic alfalfa with Bar. M, Trans2K DNA Marker; “-”, negative control; “+”, positive control; WT, wild type; number, transformation line. [file Image_2.TIF]
