# Supplementary material for: MsTHI1 overexpression improves drought tolerance in transgenic alfalfa (Medicago sativa L.)
Source: Front Plant Sci. 2022 Sep 8;13:992024. doi: 10.3389/fpls.2022.992024 (PMC9495609; doi:10.3389/fpls.2022.992024)
Supplement: Supplementary Table S1 — List of primers used in this study. [file Table_1.DOCX]

**Table S1. List of primers used in this study.**

| **Genes** | **Primer sequence (5’-3’)** |
| --- | --- |
| *MsTHI1*-F | ATGGCTTCAGCTTCCACCAC |
| *MsTHI1*-R | TTAAGCTTCTGCAATATCAGCAGA |
| *MsTHI1*-BamHI-F | CGGGGGACTCTTGACGAGCTCATGACGACGTCGTTCGCC |
| *MsTHI1*-SacI-R | CATGTCGACTCTAGAGGATCCAAATTCACCTTTTCCATTTTGGTT |
| *MsTHI1*-F (qPCR) | TGTGGCCATGATGGTCCTTT |
| *MsTHI1*-R (qPCR) | TCCTCGGCAGTGTTCATGTC |
| *GAPDH*-F | GGCTGCCATCAAGGAGGAAT |
| *GAPDH*-R | TCCAAGCTCAGCCTCATCAAG |
| *NtActin*-F | TTTGAGACTTTCAATGTGCCCGCC |
| *NtActin*-R | TAGCATGTGGGAGTGCATAACCCT |
| *CPK4*-F | GTAGAAGAAGCTGCCGGACT |
| *CPK4*-R | AATCGGGAACTTGATGGCCT |
| *CPK13*-F | TGGAGCTTTGTGAAGGTGGG |
| *CPK13*-R | AACCTCCACAATCGTCCTCG |
| *CPK21*-F | TGCAGATAACGATGGTCGGA |
| *CPK21*-R | ACAAGGTCGAATCACGCCTC |
| *THI4*-F | AAGACCAATTGCTACCCGCA |
| *THI4*-R | AACGACGGGTCATTTCACGA |
| *Cu,Zn-SOD*-F | CCGTCGGTGATGATGGAACC |
| *Cu,Zn-SOD*-R | GCATGAACAACAACAGCCCT |
| *TH1*-F | AACAGCAGGTCCAAACGCTAA |
| *TH1*-R | CCACGGAATCTCTTGCTGCC |
| *RbcX2*-F | CGTACAAGGCTGTGAGGACT |
| *RbcX2*-R | TGTTCCTTCCCAAGCAATCG |
| *TPK1*-F | AGAGCTATGCATTCTTGCCGT |
| *TPK1*-R | AGGATAATGCGCGTGTTGGA |
| *MsTHI1*-PstI-F | GAGCTCGGTACCCGGGGATCC ATGGCTTCAGCTTCCACCAC |
| *MsTHI1*-BamHI-R | CCTTTAAGCTCGACCCTGCAG TTAAGCTTCTGCAATATCAGCAGA |
| *MsTHI1*-SpeI-F | CGACTCTAGAGGATCCCCGGGATGGCTTCAGCTTCCACCAC |
| *MsTHI1*-XbaI-R | GGCGGCCGCTCTAGAACTAGTTTAAGCTTCTGCAATATCAGCAGA |
| *Hyg*-F | ATGAAAAAGCCTGAACTCACC |
| *Hyg*-R | CTATTTCTTTGCCCTCGGAC |
| *Bar*-F | ATGAGCCCAGAACGACGC |
| *Bar*-R | CAAATCTCGGTGACGGGC |
